# Bias-induced electrostatic magnetoresistance in ferromagnet/chiral systems

**DOI:** 10.1038/s41467-025-65680-5

**Published:** 2025-11-11

**Authors:** Sytze H. Tirion, Bart J. van Wees

**Affiliations:** Zernike Institute for Advanced Materials, University of Groningen, Groningen, The Netherlands

**Keywords:** Nanoscience and technology, Electronics, photonics and device physics

**arising from** Y. Zhao et al. *Nature Communications* 10.1038/s41467-024-55433-1 (2025)

In a recent paper, Zhao et al.^1^ propose an explanation for the experimentally observed magnetoresistance, known as the chirality-induced spin selectivity MR (CISS MR) of ferromagnet/tunnel barrier/(molecular)chiral systems. This is based on electrostatic barrier modification caused by charge trapping. The authors propose a non-Hermitian skin effect (NHSE) where the localization direction of the electronic wavefunctions depends on the magnetization direction and the (sign of) chirality *χ*. They argue that in this way, for a given chirality, the trapped charge depends on the magnetization direction and, therefore, modifies the barrier height when M is reversed. This changes the charge transport and thus produces a magnetoresistance (MR). The authors state that the NHSE should not be effective in equilibrium, and argue that for the NHSE to produce a magnetization-dependent charge trapping, “*current-related / current-induced dissipation*” is required. However, the authors, Zhao et al., do not explain how a current generates the charge trapping, nor how the MR will depend on the (direction of the) current. Understanding the bias dependence of the CISS MR is an outstanding issue that we specifically address here.

We agree with the authors that an electrostatic mechanism/barrier modification model^1,2^ should be considered for the CISS MR, but here we point out that:

1. A current-induced MR effect, as described by the authors, can also be generated by other models that describe coupled spin and charge transport in ferromagnet/chiral systems and resulting changes in electrostatic potentials.
2. However, independent of the model, we show below that the MR, which is generated in this way, will scale linearly with the bias current (and/or bias voltage), and the MR will change sign with bias reversal. This is, however, not observed in (most of) the experiments^3,4^.

**Fig. 1:**
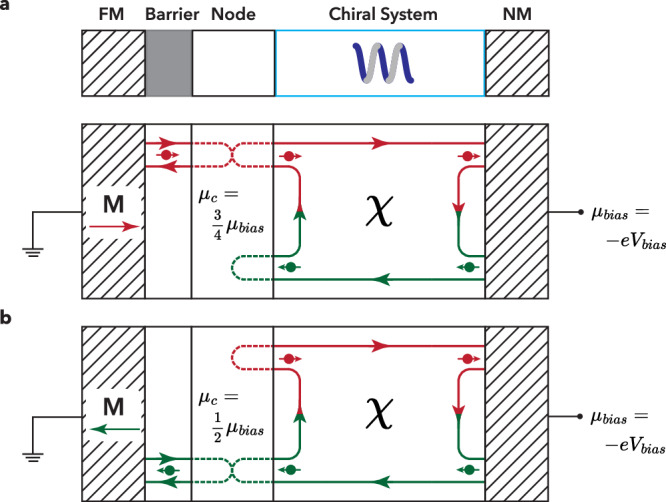
Elementary circuit representing a ferromagnet, spin-dependent tunnel barrier, node, chiral element, and counter electrode. **a** Shows the (directional) flow of (→) polarized spins (red) and (←) polarized spins (green), and **b** with the magnetization directions reversed. In addition to the spin-dependent directional transmissions, the chiral element also shows the (directional) spin-flip reflections, which are inherently part of spin charge transport in chiral systems^5,6^. A comparison of **a** and **b** shows that these configurations of M and *χ* are not equivalent, and when a bias voltage is applied, the node has a different *μ*_*c*_ as well as *μ*_*s*_ for these configurations.

To illustrate our point, we consider the elementary circuit geometry of Fig. 1. It consists of a ferromagnet with magnetization M (switched in +/− x-direction); a tunnel barrier (e.g. an oxide or vacuum) with spin-polarized tunneling, controlled by M; a node, which represents a region where charges and spins can accumulate; and a chiral system *χ*. We assume that the chiral system obeys the symmetries of chirality-induced spin selectivity (CISS) for the coupling between spin and charge electrochemical potentials and spin and charge currents^5,6^. Finally, a bias voltage *V*_*bias*_ (described by an electrochemical potential ${\mu }_{{bias}}=-e{V}_{{bias}}$) can be applied to the (non-magnetic) contact to induce a charge current. For clarity of our argument, we assume that the conductance of the tunnel junction and spin transmissions/reflections of the chiral system are 100 % spin-polarized and have the same charge conductance *G*. It was discussed in refs. ^5,6^ that even though the chiral system exhibits CISS spin-charge coupled transport, the two-terminal resistance in the linear transport regime does NOT depend on the direction of M (and/or the sign of *χ*). A calculation of the two-terminal conductance gives *G*/2 for both cases, which confirms the generality of the Onsager reciprocity relations.

However, as is implicit in refs. ^5,6^, but not discussed in detail there, the spin electrochemical potentials *μ*_→_ and *μ*_←_, and therefore also the charge and spin electrochemical potentials ${\mu }_{c}=\,({\mu }_{\to }+\,{\mu }_{\leftarrow \,})/2$ and ${\mu }_{s}=\,({\mu }_{\to }-\,{\mu }_{\leftarrow \,})/2$ in the node DO depend on the combinations of the direction of M and *χ*. Fig. 1a, b shows the nonequivalent flow of spin and charge for both combinations. A calculation shows that *μ*_*c*_ and *μ*_*s*_ change for two directions of M. As a consequence, when a *V*_*bias*_ is applied (and charge and spin currents flow) *μ*_*c*_ will scale linearly with *V*_*bias*_, as should be in the linear transport regime, and will change sign when the bias voltage/current is reversed. This will not produce an MR as long as the transport properties of both components remain unchanged in the relevant energy range.

**Fig. 2:**
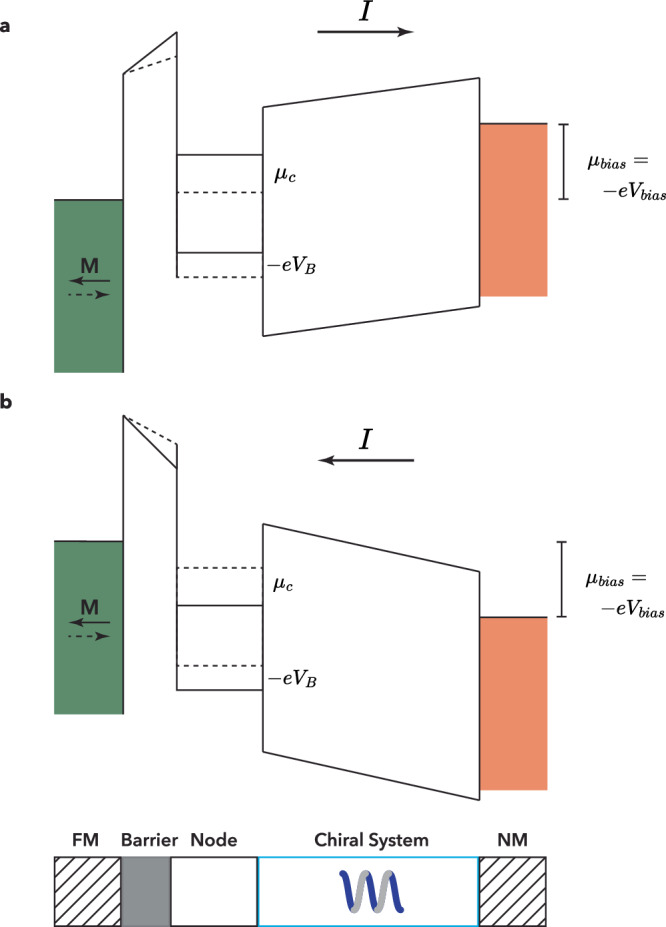
Band diagram illustrating the magnetization direction-dependent change in electrostatic potential in the presence of an applied bias voltage *V*_*bias*_ in a ferromagnet/tunnel barrier/(molecular) chiral system. **a** Shows the change in electrochemical potential *μ*_*c*_ of the node and the corresponding electrostatic energy of the bottom of the band $-e{V}_{B}$, which results in a modification for the barrier height for two different magnetization directions (indicated with the solid and dashed lines), resulting in an MR. **b** Shows the same for an opposite bias voltage. The resulting electrostatic charging is reversed, and this leads to an MR, which is also reversed.

We now show that an MR can occur when electrostatic charging is included. The number of occupied states in the node, and therefore the electronic charge, will depend on the difference between the electrostatic energy of the bottom of the band $-e{V}_{B}$, and *μ*_*c*_ (which describes up to which energy the states are occupied.) (We assume that a finite *μ*_*s*_ does not play a role). Raising *μ*_*c*_ will increase the electron density, and this will raise the electrostatic energy $-e{V}_{B}$ of the bottom of the band. The final result will depend on a self-consistent Poisson-type calculation, but this will not change the essence of the effect, $-e{V}_{B}$ will be raised. A difference in electrostatic potential $-e{V}_{B}$ will, in turn, modify the shape of the barrier, effectively lowering or raising it. (Fig. 2a, b). This will then change the (charge) conductance of the barrier, and also the two-terminal resistance experienced by that charge flow. Changing M will produce a different *μ*_*c*_, and a different electrostatic potential, and thus, a two-terminal MR can be generated.

This current-induced electrostatic charging of the node, therefore, results in a departure from (exact) linear response and will result in an MR, which will scale linearly with bias. However, this is not compatible with the observations in most experiments^3,4^, which show an (almost) bias-independent MR that does not change sign with the applied bias. The MR above actually resembles the current-induced MR of the electrical magnetochiral anisotropy (EMCA) effect^7^, but this explicitly requires going beyond the linear response regime.

Our argument above of the bias dependence of the electrostatically induced MR does not change when we introduce (localized) trapping states in the node region, which can store the charge on long timescales. The (slow) charging/discharging of these trap states will also be determined by the difference of *μ*_*c*_ and $-e{V}_{B}$. The conclusion, therefore, remains that this will depend on the (bias direction of the) current which has induced this trapping. At higher biases, deviations from the above picture can occur. Other electronic bands and localized states might be involved. However, the process of charge accumulation (or depletion) and possible charging and discharging of trap states is still reversed by changing the bias direction. Thus, still an MR, which is ODD in the applied bias, is generated. We note that we have used a specific idealized model. However, similar considerations may be applied when considering the effect of spin and charge transport on the molecular level.

In our case, the interaction between M and *χ* results from current-induced spin-dependent transport and the associated charge accumulation in the node. The authors introduce a non-Hermitian skin effect, which results from the (coherent) coupling of the spin-polarized states in the ferromagnet and the chiral system. It is not clear if this model requires spin transport. The directional and spin dependence of the wavefunction decay is a specific manifestation of the coupling of magnetism and chirality. In our opinion, this does not require the NHSE, as our model shows. However, similar to the observations above, in agreement with the Onsager reciprocity relation, the authors state that this NHSE by itself should not produce an MR (in linear response). The effect they describe is therefore also bias current induced, and should therefore have the same bias-induced characteristics as our model system above.

The authors show that they can fit experimental results with a Simmons formula where the barrier height changes with magnetization direction, but does NOT change with bias magnitude or direction. The model presented by the authors explicitly requires a bias current-induced mechanism. However, we deem it unlikely that a magnetization-dependent trapped charge is first induced by the current and then becomes current-independent when the IV characteristics are scanned over a large positive and negative bias range. The experimental observations are not compatible with a bias-induced charge (trapping) model, independent of which exact mechanism for coupling chirality and ferromagnetism (and therefore also including the model above)

In our ACS nano paper^2^ we described an electrostatic charging model based on the experimental observations of the magnetization and chirality-dependent contact potential, see refs. ^8,9^. The observation of almost perfectly linear IV-curves, which have an (almost) bias-dependent MR, is, in our opinion, more compatible with a model where the electrostatic potential does not depend on the bias current, as we have discussed in ref. ^2^. This implies that electrostatic charging already occurs in or very close to equilibrium. In agreement with the authors, we expect that there should be no effect between chirality and magnetism in or close to equilibrium, so it remains an outstanding question what is the microscopic origin of the electrostatic charging.
